# Definitive Endoderm Formation from Plucked Human Hair-Derived Induced Pluripotent Stem Cells and SK Channel Regulation

**DOI:** 10.1155/2013/360573

**Published:** 2013-04-16

**Authors:** Anett Illing, Marianne Stockmann, Narasimha Swamy Telugu, Leonhard Linta, Ronan Russell, Martin Müller, Thomas Seufferlein, Stefan Liebau, Alexander Kleger

**Affiliations:** ^1^Department of Internal Medicine I, University of Ulm, Albert-Einstein Allee 23, 89081 Ulm, Germany; ^2^Institute for Anatomy and Cell Biology, University of Ulm, Albert-Einstein Allee 11, 89081 Ulm, Germany

## Abstract

Pluripotent stem cells present an extraordinary powerful tool to investigate embryonic development in humans. Essentially, they provide a unique platform for dissecting the distinct mechanisms underlying pluripotency and subsequent lineage commitment. Modest information currently exists about the expression and the role of ion channels during human embryogenesis, organ development, and cell fate determination. Of note, small and intermediate conductance, calcium-activated potassium channels have been reported to modify stem cell behaviour and differentiation. These channels are broadly expressed throughout human tissues and are involved in various cellular processes, such as the after-hyperpolarization in excitable cells, and also in differentiation processes. To this end, human induced pluripotent stem cells (hiPSCs) generated from plucked human hair keratinocytes have been exploited *in vitro* to recapitulate endoderm formation and, concomitantly, used to map the expression of the SK channel (SKCa) subtypes over time. Thus, we report the successful generation of definitive endoderm from hiPSCs of ectodermal origin using a highly reproducible and robust differentiation system. Furthermore, we provide the first evidence that SKCas subtypes are dynamically regulated in the transition from a pluripotent stem cell to a more lineage restricted, endodermal progeny.

## 1. Introduction

Mammalian development is a tightly regulated process, with considerable biochemical and physiological changes occurring from the time of fertilization to the onset of gastrulation and further differentiation towards fully formed organisms. However, understanding early fate decision events, such as segregation of the three germ layers, is a prerequisite for regenerative medicine [1–5]. The advent of induced pluripotent stem cells and their unique features of unlimited self-renewal and nonrestricted differentiation capacity marked a milestone in the battle to dissect such processes—directly in the context of human development [6–8]. Given the incredible accordance of embryonic development *in vivo* and its respective model system *in vitro*, it is not surprising that most of the currently available pluripotent stem cell differentiation protocols make use of physiological, stage-specific signalling clues in order to recapitulate development of all three germ layers: ectoderm, mesoderm, and endoderm. Further differentiation towards more specialized cell types has also been achieved, for example, formation of primitive gut tube endoderm (SOX17/Hnf1b positive [9, 10]), pancreatic progenitor cells (Pdx1/Cpa1 positive [11, 12]), and hepatic progenitor cells (AFP/HNF4a positive [13]) from definitive endoderm progenitor cells. Nevertheless, the precise mechanisms governing such complex processes are not completely understood. Another limitation exists in achieving highly homogenous, reproducible cell type-specific yields. As a result, the current use of hiPSCs for disease modelling where the aim is to use *in vitro* differentiated patient-specific pluripotent stem cells to replace the patients' damaged cells is massively hindered. In consequence, critically defined, efficient, and robust differentiation protocols are highly anticipated. 

Endoderm comprises the innermost of the primary germ layers of an animal embryo and has a primary role to provide the epithelial lining of two major tubes within the body. The first tube, which extends the entire length of the body, is known as the digestive tube and undergoes budding during embryogenesis to form the liver, gallbladder, and pancreas. The second tube, the respiratory tube, forms an outgrowth of the digestive tube and gives rise to the lungs. Notably, two distinct sets of endoderm can be distinguished in the developing embryo: visceral endoderm arising directly from the inner cell mass and definitive endoderm (DE) derived from mesendoderm within the anterior primitive streak in close proximity to the cardiovascular progenitors [1, 14–16]. The visceral endoderm forms the epithelial lining of the yolk sac [17] while the DE is responsible for the internal (mucosal) lining of the embryonic gut and is governed by the expression of key transcription factors such as SOX17 [18], Foxa2, or Hex1 [19]. 

To date, a large group of proteins has been broadly neglected concerning its role during developmental processes, namely, ion channels. In addition to the modulation of the membrane potential in various tissues and cell populations, ion channels were identified to be involved in a number of biological processes, such as proliferation, cell differentiation, and cell morphogenesis. Since these mechanisms are apparently abundant in the transition of stem or progenitor cell populations to more defined cells types of different origin and potency, a role for ion channels in developmental processes can be hypothesized [20–23]. In particular, the adsorptive tissues derived from the DE are often rich in ion channels and defects in these channels are responsible for some harmful diseases. One prominent example is cystic fibrosis (CF), a common, autosomal recessive disorder due to mutations in a chloride channel known as the CFTR. Located on the plasma membrane of many epithelial cells, this simple mutation gives rise to abnormalities of salt and fluid transport in many endodermal derived tissues including lung, pancreas, and liver [24]. However the contribution of other ion channel families to diseases within the foregut has been poorly studied. 

Indeed, in pluripotent stem cells, activation of small and intermediate conductance calcium activated potassium channels (SK channels; SKCas) triggers the MAPK/ERK pathway following RAS/RAF activation finally, giving rise to cytoskeletal rearrangement, cardiogenesis, and cardiac subtype specification [2, 3, 5, 25]. The group consists of four members, namely, SK1 (KCa 2.1, KCNN1), SK2 (KCa 2.2, KCNN2), SK3 (KCa 2.3, KCNN3), and SK4 (KCa 3.1, KCNN4). The functional form of the ion pore is mediated by the combination of the 4 subunits, respectively. Additionally, widely distributed functional splice variants of SKCas have been found throughout the organism in several tissues [26–28]. Functional SKCas are not only constructed as homo- but also as hetero-tetrameric channel proteins, most probably serving a cellular and functional specificity [26, 29]. The pore is opened following subtle elevation of intracellular calcium levels. Calmodulin, attached in a Ca^2+^-dependent manner to the C-terminal of the channel subunits, specifically binds Ca^2+^-ions and mediates a conformational change of the channel protein, leading to the opening of the pore [30, 31]. Calcium is the only known physiological activator of SKCas and channel opening occurs within a few milliseconds [31]. SK1-3 are highly expressed in the nervous system where they modify the membrane potential; that is, they crucially contribute to the after-hyperpolarization and therefore regulate the firing pattern, frequency, and length of action potentials in different neuronal networks [32–35]. On the other hand, SKCas play important roles in multiple other cellular functions, namely in cerebral and peripheral blood vessel smooth muscle, the functional myocardium, or neural progenitor cells [21, 36, 37].

In the current study, we highlight a robust and efficient differentiation protocol to drive plucked human hair-derived hiPSCs towards definitive endoderm. Furthermore, we analyse changes in protein and mRNA expression in the SKCa family of ion channels in the transition from a pluripotent cell state to a definitive endodermal committed cell type. 

## 2. Materials and Methods

### 2.1. Keratinocyte Culture from Plucked Human Hair

Outgrowth of keratinocytes from plucked human hair was induced as described previously [25, 38]. Keratinocytes were split on 20 *μ*g/mL collagen IV-coated dishes and cultured in EpiLife medium with HKGS supplement (both Invitrogen, USA). The use of human material in this study has been approved by the ethical committee of the Ulm University (Nr. 0148/2009) and in compliance with the guidelines of the Federal Government of Germany and the Declaration of Helsinki concerning Ethical Principles for Medical Research Involving Human Subjects.

### 2.2. Rat Embryonic Fibroblasts (REFs) Culture

REFs were isolated from day E14 Sprague Dawley rat embryos as described previously [38] and cultured in DMEM supplemented with 15% FCS, 2 mM GlutaMAX, 100 *μ*M nonessential amino acids, and 1% Antibiotic-Antimycotic (all Invitrogen). Cells were passaged using 0.125% trypsin digestion when reaching confluence for up to 5 passages. All animal experiments were performed in compliance with the guidelines for the welfare of experimental animals issued by the Federal Government of Germany, the National Institutes of Health, and the Max Planck Society (Nr. O.103).

### 2.3. Lentivirus Generation

Lentivirus containing a polycistronic expression cassette encoding OCT4, SOX2, KlF4, and cMYC was produced as described previously [3, 25, 38].

### 2.4. Reprogramming Keratinocytes

Keratinocytes at 75% confluence were infected with 5 × 10^5^ proviral genome copies in EpiLife medium supplemented with 8 *μ*g/mL polybrene on two subsequent days. On the third day, keratinocytes were transferred onto irradiated REF feeder cells (2.5 × 10^5^ cells per well irradiated with 30 Gy). Cells were cultured in hiPSCs medium in a 5% O_2_ incubator and medium was changed daily. After 3–5 days small colonies appeared, showing a typical hiPSCs like morphology. Around 14 days later, hiPSC colonies had the appropriate size for mechanically passaging and were transferred onto irradiated MEFs or onto Matrigel-coated (BD, USA) dishes for further passaging.

### 2.5. hiPSC Culture

hiPSCs were initially cultured on feeder cells in Knockout DMEM (Invitrogen) supplemented with 20% Knockout Serum Replacement (Invitrogen), 2 mM GlutaMAX (Invitrogen), 100 *μ*M nonessential amino acids (Invitrogen), 1% Antibiotic-Antimycotic (Invitrogen), 100 *μ*M *β*-mercaptoethanol (Millipore, USA), 50 *μ*g/mL ascorbic acid, and 10 ng/mL FGF2 (both PeproTech, USA) in a 5% CO_2_ incubator.

For later passages, hiPSCs were mechanically picked and transferred onto Matrigel-coated dishes (BD) and kept in FTDA medium that contains DMEM/F12-GlutaMax (Invitrogen), 1 × ITS (Insulin-Transferrin-Selenium, Invitrogen), 0.1% HSA (Biological Industries, Israel), 1 × Lipid mix (Invitrogen), 1 × Penicillin-Streptomycin (Millipore), 10 ng/mL FGF2 (PeproTech), 0.5 ng/mL TGF-*β*1 (PeproTech), 50 nM Dorsomorphin (Sigma, Germany), and 5 ng/mL Activin A (PeproTech) [39]. hiPSCs were cultured in a 5% O_2_ incubator and medium was changed daily. For splitting cells, hiPSCs colonies were incubated with Dispase (StemCell Technologies, France) for 5–7 min at 37° and subsequently detached using a cell scraper. After brief centrifugation, cells were resuspended in FTDA medium and transferred onto Matrigel-coated dishes. Excision of the viral cassette was achieved by incubation with recombinant TAT-Cre protein (1.5 *μ*M for 5 h). 

### 2.6. Monolayer-Based hiPSC Differentiation towards Definitive Endoderm

For DE differentiation, hiPSCs were plated onto Matrigel-coated dishes and cultured in FDTA medium supplemented with 10 *μ*M Rock-inhibitor Y-276342 (Ascent, UK) for 24 hours. When the cells reached about 75% confluence, medium was changed to RPMI 1640 medium (Invitrogen) containing 2% FBS (Lonza, CH) with 500 nM IDE1 (R&D systems, USA), 3 *μ*M CHIR99021 (Axonemedchem), 5 *μ*M LY294002 (Sigma, Germany), and 10 ng/mL BMP4 (PeproTech) for 24 hours. Then, medium was changed to RPMI 1640 medium containing 2% FBS and supplemented with 500 nM IDE1 and 5 *μ*L LY294002 for two days. From day 3 on, cells were cultured in RPMI 1640 supplemented with 500 nm IDE1, 5 *µ*M LY294002, and 50 ng/mL FGF2. The respective figure contains an experimental outline illustrating detailed culture conditions and treatment regimens [40–45] (Figure 2(a)). 

### 2.7. Immunocytochemistry

Immunofluorescence has been previously described [5]. Nuclei were stained with DAPI. Primary antibodies were used as follows: SK1/2 (both 1 : 100, Sigma), SK3 (1 : 100, Alomone Labs, Israel), SK4 (1 : 100, Cell Applications, USA), SOX17 (1 : 500, R&D systems), and FOXA2 (1 : 100, Santa Cruz, USA). hiPSC lines were characterized using the StemLite Pluripotency Antibody Kit (Cell Signaling, USA). Fluorescence labelled secondary antibodies were Alexa Fluor 488 and Alexa Fluor 568 (both Invitrogen). Images were captured using an upright fluorescence microscope (Axioskop 2, Zeiss, Oberkochen, Germany) equipped with a Zeiss CCD camera and analysed using Axiovision software (Zeiss) [46].

### 2.8. Quantitative One-Step Real Time

RT-PCR (qPCR) Analysis was performed as previously described. Briefly, one-step real-time qPCR was carried out with the LightCycler System (Roche, Mannheim, Germany) using the QuantiTect SYBR Green RT-PCR kit (Qiagen, Hilden, Germany). Relative transcript expression was expressed as the ratio of target gene concentration to the housekeeping gene hydroxymethylbilane synthase (*HMBS*) [47, 48].

### 2.9. FACS Analysis 

For flow cytometry cells were harvested with TrypLE (Invitrogen) for 7 min at 37°C to obtain single-cells suspension. Next, cells were washed twice with PBS, blocked with 5% HSA-solution (in PBS) to avoid unspecific binding of the antibodies to the Fc-receptor. Cells were washed again with PBS and incubated for 40 min at 4°C with CXCR4-PE (Invitrogen), subsequently c-Kit-APC (Invitrogen) was added for additional 10 min at 4°C in FACS buffer (2% FCS in PBS), according to the manufacturer's instructions. Cells were washed with FACS buffer, 50 ng/mL DAPI was added, to exclude dead cells from analysis, and the samples were directly analysed on a LSRII flow cytometer (BD).

For intracellular SOX17 staining cells were washed twice with PBS, blocked with 5% HSA-solution (in PBS) to avoid unspecific binding of the antibodies to the Fc-receptor. Cells were washed again with PBS and the pellet was resuspended in 4% PFA and incubated for 15 min at 37°C for fixation. Subsequently the cell pellet was resuspended in 0.5% Saponin in FACS buffer (saponin buffer) and incubated for 30 min on ice. Cells were pelleted and stained with SOX17 (1 : 100, R&D systems) at 4°C for one hour. Cells were washed with saponin buffer and afterwards incubated for 30 min at 4°C with anti-goat Alexa Fluor 647. Finally cells were washed with FACS buffer and directly analysed on an LSRII flow cytometer (BD).

### 2.10. Statistical Analysis

If not stated otherwise, error bars indicate standard deviations. Calculations were done with GraphPad Prism 5 (GraphPad Software, Inc., San Diego, http://www.graphpad.com/).

## 3. Results

### 3.1. Reprogramming Human Hair-Derived Keratinocytes to hiPSCs

**Figure 1:**
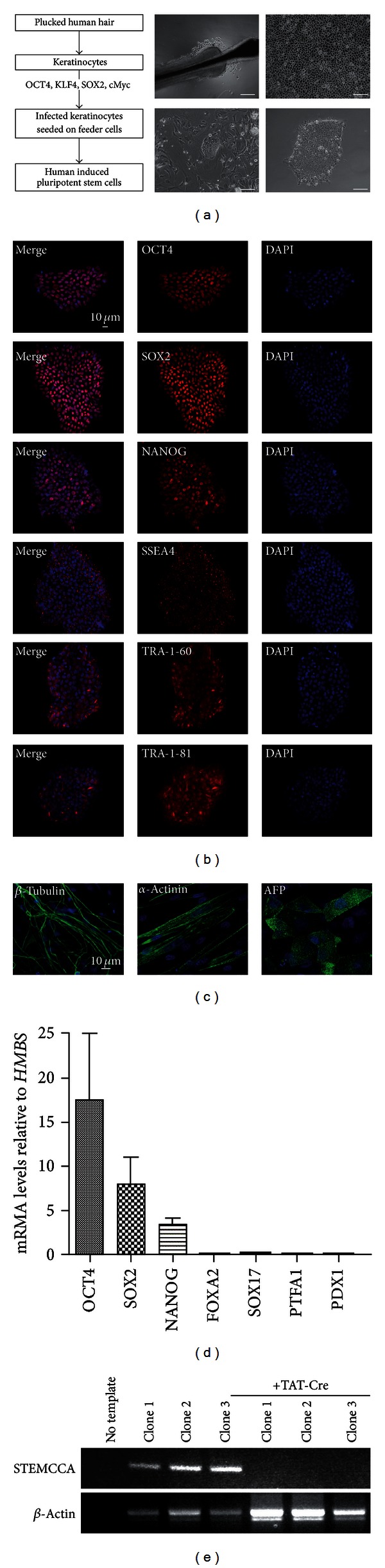
Generation of human induced pluripotent stem cells. (a) Scheme of reprogramming keratinocytes from human plucked hair into induced pluripotent stem cells (hiPSCs). Bright field microscopy images from outgrowing keratinocytes, in detail, the outer root sheath of a plucked human hair. Keratinocytes were infected with a lentiviral construct containing the reprogramming factors OCT4, SOX2, KlF4, and cMyc on two subsequent days. On the following day keratinocytes were transferred onto a monolayer of irradiated REFs (rat embryonic fibroblasts) and after some days small hiPSC colonies could be detected. For later passaging hiPSCs were cultured, under feeder-free conditions, on Matrigel-coated dishes in FTDA medium. Scale bars are 20 *μ*m. (b) hiPSCs express the nuclear factors OCT4, SOX2, and NANOG as well as the pluripotent surface markers SSEA4, TRA-1-60, and TRA-1-81 (*all red*). Scale bars as indicated. (c) hiPSCs used in the present study are capable of differentiating into cells of all 3-germ layers represented by *β*-tubulin (beta-tubulin 3 in green, neurons—ectoderm), *α*-actinin (alpha-actinin in green, myocytes—mesoderm), and AFP (alpha-fetoprotein in green, liver cells—endoderm). Nuclei are stained with DAPI in blue. (d) Transcript levels of pluripotent markers such as *OCT4*, *SOX2,* and *NANOG* were highly expressed whereas markers for definitive endoderm (*SOX17* and *FOXA2*) and markers for pancreatic progenitor cells (*PTFA1* and *PDX1*) were not expressed at all. (e) Polymerase chain reaction to detect the STEMCCA cassette in iPS cell subclones before and after treatment with recombinant Cre protein (actin band ~480 bp, STEMCCA band ~400 bp).

For the depicted studies we utilized keratinocyte cultures from plucked human hair of healthy individuals (Figure 1(a)). With the use of a lentiviral, multicistronic four-factor reprogramming system, keratinocytes were successfully reprogrammed to human induced pluripotent stem cells displaying embryonic stem cell like morphology (Figure 1(a)) as well as hallmarks of pluripotency tested via immunohistochemistry and qRT-PCR for the expression of embryonic stem cell markers. Several lines of more than 5 individuals (data not shown or reported in [3, 25, 38]) were tested for their proliferation and differentiation capacity and subsequently two lines were selected, named “hiPSC_1 and hiPSC_2.” Both lines were additionally tested for the protein expression of OCT4, SOX2, NANOG, SSEA4, TRA1-60, and TRA1-81 (Figure 1(b)) and mRNA levels of three pluripotency markers (*OCT4*, *SOX2*, *NANOG*). At the pluripotent stage definitive endoderm makers (*FOXA2*, *SOX17*) and markers for pancreatic progenitors (*PTFA1*, *PDX1*) were negative (Figure 1(d)). Additionally, all lines were capable of differentiating into cells of all 3 germ-layers, as shown by *β*-3-tubulin (neurons—ectoderm), *α*-actinin (muscle cells—mesoderm), and *α*-fetoprotein (liver cells—endoderm) (Figure 1(c)). One line was further treated with recombinant Cre protein to excise the reprogramming STEMCCA cassette being flanked with loxp sites. To test for successful excision, PCR amplification of the STEMCCA cassette was performed in cre- and nontreated iPS cell clones from this respective line showing the band only in controls (Figure 1(e)). Taken together, our established hiPSC lines display an embryonic stem cell like phenotype, proven by morphology and expression of pluripotency markers as well as absent mRNA for endodermal markers.

### 3.2. Human Induced Pluripotent Stem Cells Can Be Differentiated to Cells Representing the Definitive Endoderm

**Figure 2:**
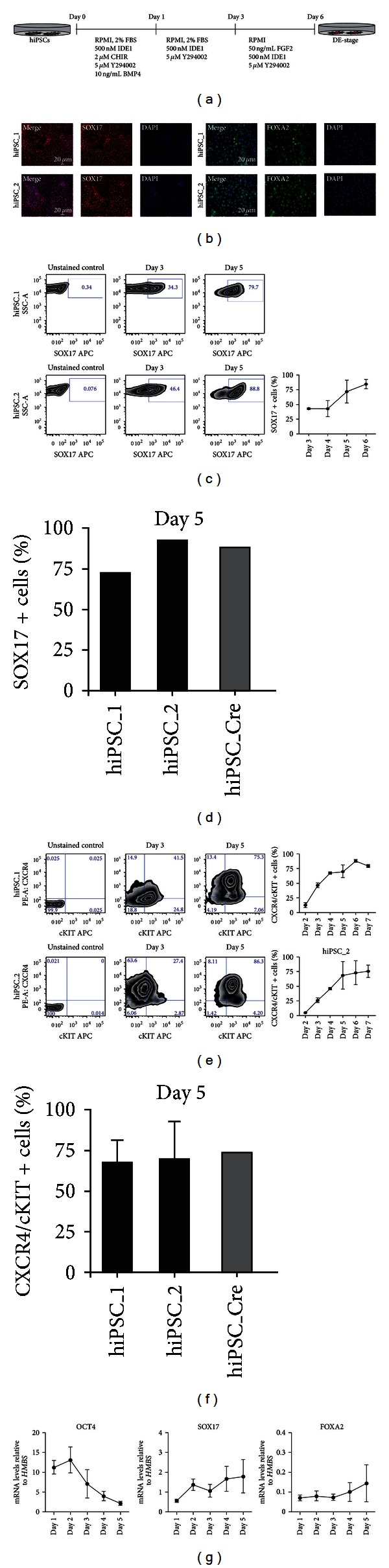
Differentiation of hiPSCs into DE (definitive endoderm) cells. (a) Scheme of monolayer differentiation of hiPSCs into DE cells. (b) Immunocytochemistry shows that hiPSCs-derived DE cells express the early endodermal markers SOX 17 (*red*) or FOXA2 (*green*). Scale bars as indicated. (c) FACS analysis reveals that after 3 days of differentiation approximately 45% of the cells have become SOX17 positive. After 6 days of differentiation about 80% of the cells express SOX17. This data was consistent within two different hiPSC lines. (d) (e) Number of CXCR4/cKIT double-positive cells after 3 days and 5 days of differentiation, respectively. After 5 to 6 days of differentiation both hiPSCs lines express the highest amount of CXCR4/cKIT double-positive cells in the region of 75–80%. (f) Virus-containing hiPSCs (hiPSC_1/hiPSC_2) did not show differences in the number of CXCR4/cKIT double-positive cells compared to virus-free iPSCs (hiPSC_Cre). (g) Continuous loss of *OCT4* mRNA levels during DE differentiation. In contrast, transcript levels of early endodermal genes such as *SOX17* and *FOXA2* steadily increased and reached highest levels after 5 days of differentiation. Expression levels are shown relative to the housekeeping gene *HMBS* (*n* = 4, two different hiPSCs lines).

To test the differentiation potential of our established hiPSC lines into definitive endoderm (DE), previously published protocols were combined in terms of a small molecule-driven approach [43–45]. Small molecule-based assays are less biased by batch-to-batch variations and are usually more cost effective. Upon extensive testing of different combinations, our protocol led to the following replacements of established growth factors being known to drive definitive endoderm differentiation: CHIR90021 replaced Wnt3a [40], IDE1 replaced Activin A [41], and LY294002 inhibited the AKT signalling pathway to abolish pluripotency [42]. Figure 2(a) represents a detailed scheme of the differentiation conditions used for the formation of DE from day 0 (undifferentiated pluripotent hiPSCs) to day 6 (definitive endodermal cells). *In vitro* differentiated hiPSCs became positive for endodermal markers, confirmed by positive immunostaining of cells on day 5 for FOXA2 and SOX17 (Figure 2(b)). To analyse and characterize the SOX17 expression more objectively, we quantified SOX17 expression via intracellular FACS analysis in a time course from day 3 to day 6 of the applied protocol.  Figure 2(c) shows representative FACS plots from both lines, representing SOX17 positive cells on day 3 and day 5. We did not observe differences in the differentiation capacity of virus-free hiPSCs after excision of the reprogramming cassette in comparison to virus-containing cells, making further analyses of silencing of exogenous factors unnecessary (Figure 2(d)). In summary, SOX17 expression is increasing from approximately 45% at day 3 to nearly 80% of SOX17 positive cells on day 6. Recent publications depict CXCR4 and c-KIT positive cells as definitive endoderm progenitors, that give rise to self-renewing endodermal progenitor cells (EPCs) [49]. To confirm our protocol, we did time course analysis by flow cytometry for CXCR4 and c-KIT positive cells during differentiation.  Figure 2(e) shows representative FACS plots of CXCR4 and c-KIT positive cells of the two hiPSC lines on day 3 and 5. Two independent experiments for each line were summarized and shown from day 2 to 7 of endodermal differentiation. From day 2 on, the double positive population (CXCR4 and c-KIT) is steadily increasing in both lines. The maximum is reached with almost 90% double positive cells for hiPSC_1 and almost 80% for hiPSC_2 (Figure 2(d)). Again there was no relevant difference upon excision of the reprogramming cassette (Figure 2(f)).

To further confirm the definitive endodermal identity of the differentiated lines, we measured mRNA levels using qRT-PCR analysis for OCT4, SOX17, and FOXA2. From day 1 to day 5 mRNA levels for the pluripotency marker OCT4 decrease continuously (Figure 2(g), summarized for hiPSC 1 and hiPSC 2). *SOX17* and *FOXA2* levels were tested in the two established hiPSC lines during differentiation and displayed increasing mRNA levels from day 1 to day 5 (Figure 2(g)). This data clearly indicates that the investigated hiPSC lines can be differentiated into DE, loosing markers of pluripotency and up regulating the expression of endodermal markers during endoderm formation.

### 3.3. Expression of Calcium-Activated Potassium Channels (SKCas) during DE Differentiation

**Figure 3:**
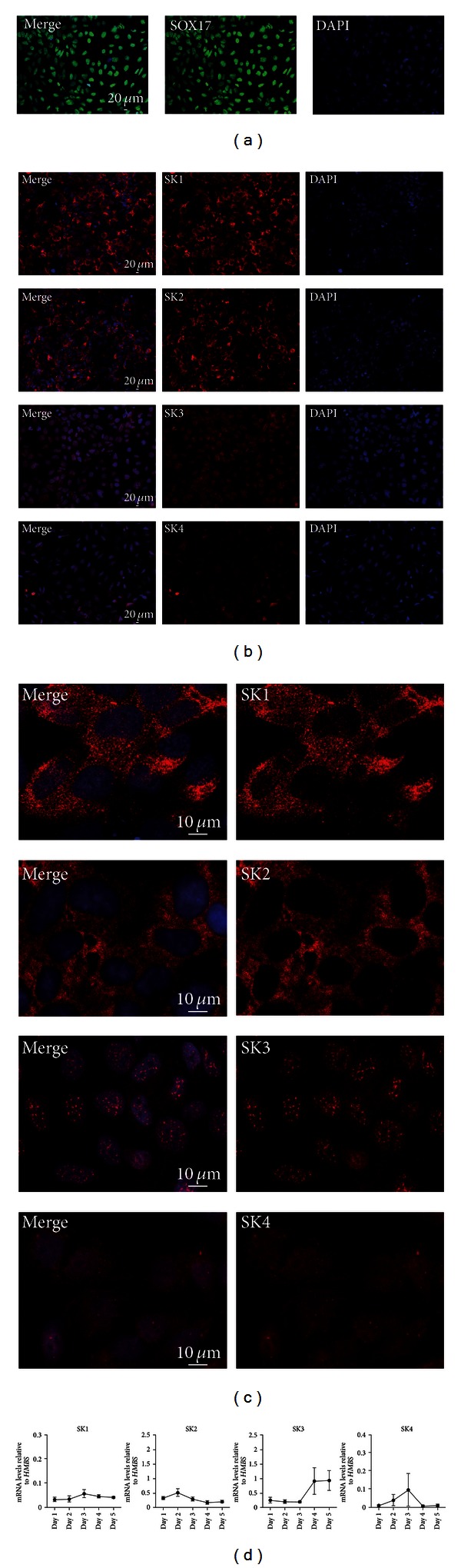
Expression of Calcium-activated Potassium channels during formation of definitive endoderm. (a) Expression of SOX17 (*green*) after 5 days of DE differentiation. (b) Immunofluorescence analysis of SKCa proteins in DE cells. Indicated SKCa subtype (*red)*. Scale bars as indicated. (c) Higher magnifications of indicated SKCa subtype (*red)*. Scale bars as indicated. (d) Transcript levels of SK1 and SK2 remained relatively low during the DE differentiation. In contrast, mRNA levels of SK3 increased after 4 days of differentiation. SK4 mRNA levels slightly increased during the first days of differentiation and peaked on day 3 followed by a sharp decrease until day 5. Expression levels are shown relative to the housekeeping gene *HMBS* (*n* = 4, two different hiPSCs lines).

Next, we had a closer look on the expression of the different SKCas subtypes during DE differentiation. hiPSCs were differentiated into DE cells and expression of SKCa was investigated after 5 days of differentiation. On day 5 SOX17 is strongly expressed indicating the differentiation into DE cells (Figure 3(a)). To analyse the expression of the SKCas, DE cells were stained for the different SKCa subtypes. Immunofluorescence analyses show a quite strong and stable expression of SK1, SK2, and SK3 whereas SK4 seems to be expressed at a lower level (Figure 3(b)). SK1, 2, and 4 are localized in the cytoplasm and the cell membrane. However, SK3 is not only localized at the cell membrane but also as PUNCTUA in the nuclei (Figure 3(c)). This is a finding that needs to be analysed in further studies. Double immunofluorescence staining for SOX17 and respective channel proteins are shown in Supplementary Figure 1 available online at http://dx.doi.org/10.1155/2013/360573. 

mRNA expression analysis via quantitative RT-PCR (qRT-PCR) shows a relative constant expression of SK1 and SK2 during DE differentiation (Figure 3(d)). In contrast, transcript levels of SK3 obviously increased after 4 days of differentiation (Figure 3(d)). SK4 mRNAs levels marginally increased during the first days of differentiation and peaked on day 3, followed by a sharp decline up to day 5 (Figure 3(d)). To note, all four SKCa subtypes are expressed during DE differentiation. SK1 and SK2 are constantly expressed whereas SK3 seems to be up regulated during ongoing DE differentiation. The transcript levels of the different SKCa subtypes on day 5 reflect our observations of the immunofluorescence analysis. In sum, all 4 SK subtypes are differentially expressed during DE differentiation of human induced pluripotent stem cells with a yet undescribed localization of SK3 in the nucleus.

## 4. Discussion

In the current study, we provide proof of the concept that plucked human hair-derived iPSCs are highly potent in their capacity to commit not only towards mesoderm [3] and neuroectoderm [25] but also towards the endodermal germ layer, particularly definitive endoderm. To this end, a newly adopted protocol based on previously published studies was applied and resulting cells were extensively characterized by gene expression analysis, immunofluorescence microscopy, and FACS-staining for intracellular and surface markers defining the definitive endoderm signature. 

As induced pluripotent cells are currently considered to resemble human embryonic stem cells, a state-of-the-art assay for hiPSC generation is required. Such an assay requires the following prerequisites: (i) noninvasive harvest of the cell type of origin, (ii) broad applicability in terms of guided differentiation to all three germ layers, (iii) useful for large-scale hiPSC biobanking, (iv) highly efficient, and (v) fast reprogramming to the hiPSC stage. Keratinocytes from the outer root sheath of plucked human hair represent such a cell source and thus points towards the generation of patient-specific human induced pluripotent stem cells as a new paradigm for modelling human disease and for individualizing drug testing. Previously, we have further optimized this method in terms of efficiency and speed by using rat embryonic fibroblasts as a feeder layer for keratinocyte reprogramming [38]. The arising hiPSCs fulfilled all the prerequisites of pluripotency including teratoma formation and spontaneous three-germ layer differentiation. 

In further studies, we have applied plucked hair-derived hiPSCs to guide differentiation towards motoneurons [25] and cardiac pacemaker cells [3], both representing highly specified cell types from either ectodermal or mesodermal origin. However, their differentiation capacity to give rise to definitive and primitive gut tube endoderm remained elusive. While forming, definitive endoderm is incorporated by morphogenetic movements into a primitive gut tube stage. This in turn is patterned into foregut, midgut, and hindgut to form the functional epithelial compartment of multiple internal organs: liver, intestines, lungs, and the pancreas [50]. Nowadays, virtually every cell population arising from the primitive gut tube has been generated using guided differentiation of pluripotent cells towards liver, intestines, lungs, and the pancreas [51–53]. Thus, the induction of DE cells marks a prerequisite for the entire process of pluripotent stem cell differentiation into, for example, pancreatic or hepatic progenitor cells [54, 55]. Several protocols have been developed and modified to increase the efficiency of DE commitment. All these protocols are strongly dependent on high doses of TGF*β* signalling mediated by Activin A as the major driving force of the process. However, large-scale differentiation experiments should be cost effective, thus, making a small molecule-based assay more desirable. To this end, we combined several previously described strategies. First, we replaced Activin A by IDE1, a compound having shown to display similar but also superior characteristics compared to Nodal or Activin A [41]. Similarly, we substituted Wnt3a by the small molecule CHIR90021 that inhibits GSK 3 kinase to mimic Wnt signalling [40]. The third small molecule LY294002 inhibited the AKT signalling pathway, by repressing PI3K, to promote the exit from pluripotency [42]. In consequence, a robust and reproducible assay was developed which shows to be effective in several human plucked hair-derived iPSCs. As the formation of definitive endoderm is a prerequisite to obtain, for example, relevant numbers of pancreatic *β*-cells, our data in combination with the presented reprogramming strategy are highly relevant for human disease modelling approaches. 

However, several studies have suggested that *β*-cells generated from human pluripotent stem cells lack adult, and at the most reach, fetal maturity as particularly expressed by their polyhormonality. This observation reinforces the notion that establishing culture conditions that promote appropriate maturation represents a significant obstacle for the generation of functional *β*-cells *in vitro* [56]. A recent landmark paper identified self-renewing definitive endodermal progenitor cells as a potential cell source to bypass this limitation. *β*-cells generated from these cells showed features of adult maturity as even shown by functional assays [57]. Given the fact that all published protocols so far lack this feature, the quality of the definitive endodermal intermediate seems to have an impact on the final maturity. The generation of definitive endodermal progenitor cells was characterized by high positivity for c-KIT and CXCR4 [57]. Thus, we included in our current DE analysis an FACS-based tool and indeed succeeded in obtaining a pattern likely to allow the isolation of this distinct cell type. The similar differentiation capacity of all our analysed plucked human hair-derived iPSCs is relevant to the field of disease modelling, using patient-specific material. Plucked hair keratinocytes are more or less the only cell type, which matches the above criteria. Nevertheless, a potential ectodermally biased epigenetic memory could limit their utility [58]. Our finding abolishes such a bias at least based on the number of different cell lines and the reproducible endodermal commitment pattern. 

The development of *in vitro* models underlying embryonic development is a prerequisite to build new knowledge and to develop new strategies targeting various genetic diseases. The development and investigation of endoderm-derived cells are such as pancreatic cells, are of high importance for the field of developmental biology and clinical implications. Induced pluripotent stem cells (iPSCs) with their unique features of unlimited self-renewal and nonrestricted differentiation capacity are a highly promising tool for regenerative medicine as well as for studies on developmental biology. iPSCs have been generated from a variety of different cells types originating from all three-germ layers [38, 58, 59]. Finally, this setup has been used to determine the expression pattern of a certain ion channel family which has been previously shown to be differentially regulated in embryonic stem cells and involved in differentiation processes, namely, small and intermediate conductance calcium-activated potassium channels [2, 3, 5, 60]. Thus, our study gives novel insights into guided pluripotent stem cell differentiation towards definitive endoderm and a potentially involved protein family. 

SKCas either exhibit small (SK1, KCa2.1, Kcnn1; SK2, KCa2.2, Kcnn2; and SK3, KCa2.3, Kcnn3) or intermediate (SK4, IK, KCa3.1, and Kcnn4) unitary conductance for K^+^ ions. Important roles in multiple cellular functions, for example, cell cycle regulation in cancer cells [20, 61], smooth muscle relaxation [23, 62], mesenchymal stem cell proliferation [22], and cytoskeleton reorganization in neural progenitors [21] have been reported. SKCas are widely expressed throughout all different tissues. While SK1 is exclusively expressed in the central nervous system, SK2 is more widely expressed in different organs arising from different germ layers such as brain, liver, or heart. SK3 is the most widespread expressed isoform with a predominant expression pattern in the central nervous system but also in smooth muscle rich tissue. SK4 can be detected in inflammatory cell-rich, surface-rich, and secretory tissues such as the pancreas [63]. In the pancreas, for example, SK4 regulates glucose homeostasis and enzyme secretion of acinar cells [64, 65]. Moreover, SKCas are overexpressed in a variety of cancers, including pancreatic cancer [20] and, for example, SK3 was shown to be involved in cancer cell migration [66]. Nevertheless, the role of SKCas in developmental processes remains enigmatic though it is well accepted that cell differentiation and maturation affect the expression patterns of ion channels. Our group has shed for the first time light on their role in differentiating pluripotent stem cells derived from mouse and men [2, 3, 5, 25]. A potential role of SKCas was already suggested by their differentially regulated expression pattern. In fact, it temporally coincides with the commitment of the cardiovascular progenitor showing an expression peak of the respective isoform around day 5 [5]. In consequence, we aimed to address the expression pattern of SKCas in the developing endoderm using plucked human hair-derived iPSCs as a bona fide modelling system. Interestingly, the differential regulation of most SKCa isoforms was relatively modest. Albeit SK2 and SK4 show a slight expression peak around day 2/3, the only regulated isoform seems to be SK3 showing a continuously increasing expression with ongoing DE formation. Interestingly, reports showing SK3 expression in DE-derived organs are restricted to a handful of studies showing SK3 expression in epithelial cancer cells and a liver-specific splice variant [67, 68]. Further studies including gain and loss of function approaches within the same assay have to clarify the respective functions of SKCa isoforms with DE formation and later maturation processes towards liver and pancreas.

To summarize, we present an efficient, novel, and robust DE formation assay being suitable for ectoderm-derived plucked human hair iPSCs. Given the prerequisites for reprogramming fulfilled by plucked human hair, a robust DE assay for this particular iPSCs type is highly relevant for disease modelling approaches. Subsequently, we have identified dynamic expression of the SKCa family of proteins during DE formation. 

## Supplementary Material

Immunofluorescence analysis of Calcium-activated Potassium Channels during definitive endoderm differentiation
