# Supplementary material for: Definitive Endoderm Formation from Plucked Human Hair-Derived Induced Pluripotent Stem Cells and SK Channel Regulation
Source: Stem Cells Int. 2013 Apr 16;2013:360573. doi: 10.1155/2013/360573 (PMC3654369; doi:10.1155/2013/360573)
Supplement: Supplementary file 1 — Immunofluorescence analysis of Calcium-activated Potassium Channels during definitive endoderm differentiation [file 360573.f1.pdf]

Supplementary Figure 1

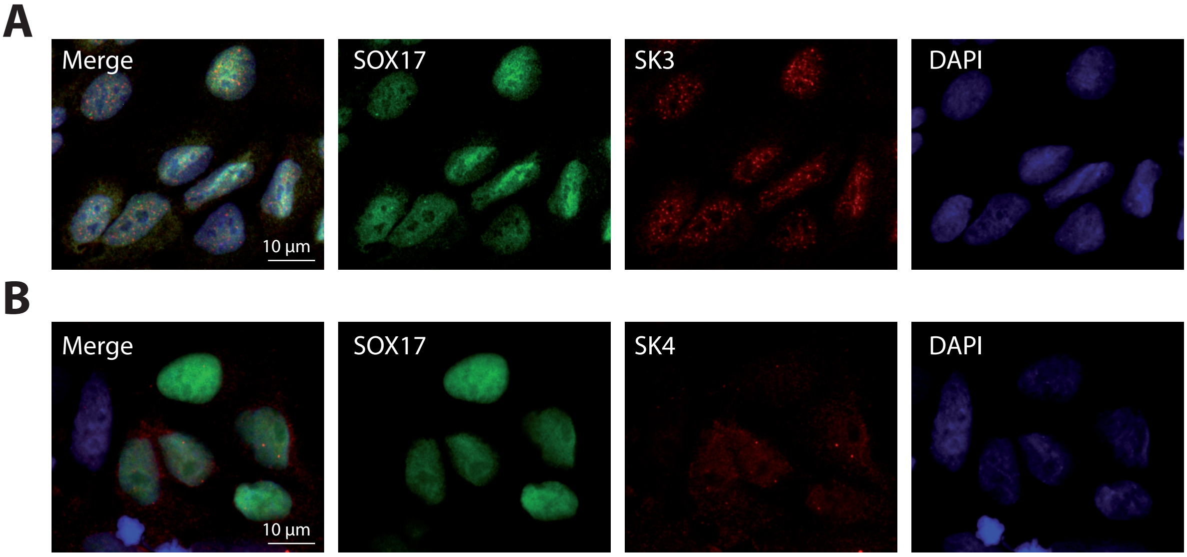

Supplementary Figure 1 Immunoflourescence analysis of Calcium-activated Potassium Channels during definitive endoderm differentiation. (A, B) Expression of SOX17 (*green*) and SK3 or SK4 (red) after 5 days of DE differentiation.
